# Conjunctival cystectomy assisted by pattern scan laser photocoagulation

**DOI:** 10.1186/s40779-017-0132-7

**Published:** 2017-07-10

**Authors:** Hee Kyung Yang, Moosang Kim, Seung-Jun Lee, Sang Beom Han, Joon Young Hyon, Won Ryang Wee

**Affiliations:** 1 Department of Ophthalmology, Seoul National University Bundang Hospital, Seoul National University College of Medicine, Seongnam, Republic of Korea; 2 Department of Ophthalmology, Kangwon National University Hospital, Kangwon National University Graduate School of Medicine, Chuncheon, Republic of Korea; 3 Department of Ophthalmology, Seoul National University Hospital, Seoul National University College of Medicine, Seoul, Republic of Korea

**Keywords:** Conjunctival cyst, Laser, PASCAL photocoagulation

## Abstract

**Background:**

To introduce a new technique of laser-assisted conjunctival cystectomy using pattern scan laser (PASCAL) photocoagulation.

**Case presentation:**

A 50-years-old Asian woman presented with a conjunctival cyst in the left eye. Slit-lamp examination revealed a 5 mm × 2 mm sized freely movable conjunctival cyst. After a 1 mm × 1 mm sized conjunctival opening was made using PASCAL photocoagulation, the cyst was extracted using a non-toothed forceps without rupture. Two weeks later, complete re-epithelialization of the conjunctiva was observed without any complications. No evidence of recurrence was noted over the 6-month follow-up period.

**Conclusion:**

Conjunctival cystectomy with the adjunctive use of PASCAL photocoagulation can be an effective and safe treatment method.

## Background

Conjunctival cysts typically develop after ocular surgery or trauma but can also arise spontaneously [1]. Although the condition is often asymptomatic, it can occasionally cause foreign body sensation or ocular discomfort [2]. Simple aspiration is often attempted as the first-line treatment [3], but recurrence occurs frequently because the epithelial cells lining the cyst capsule are not removed [1, 3]. For complete removal of the cyst and prevention of recurrence, various methods have been developed, such as simple resection [1]; surgical excision with visualization of the cyst capsule using tissue dyes, including indocyanine green or trypan blue dye [1, 4]; thermal cautery [5]; intracyst injection of sclerosing agent [6]; cyst rupture using yttrium aluminum garnet (YAG) laser [7]; argon laser photoablation [3]; and high-frequency radio-wave electrosurgery [2].

Pattern scan laser (PASCAL; OptiMedica, Santa Clara, CA, USA) has numerous advantages, including safety, effectiveness and reduced pain due to shorter exposure time. Thus, PASCAL has replaced conventional argon laser in many ophthalmological clinics. We recently experienced a case of a conjunctival cyst that was successfully removed with the adjunctive use of PASCAL photocoagulation. The case is described in this report.

## Case presentation

**Fig. 1:**
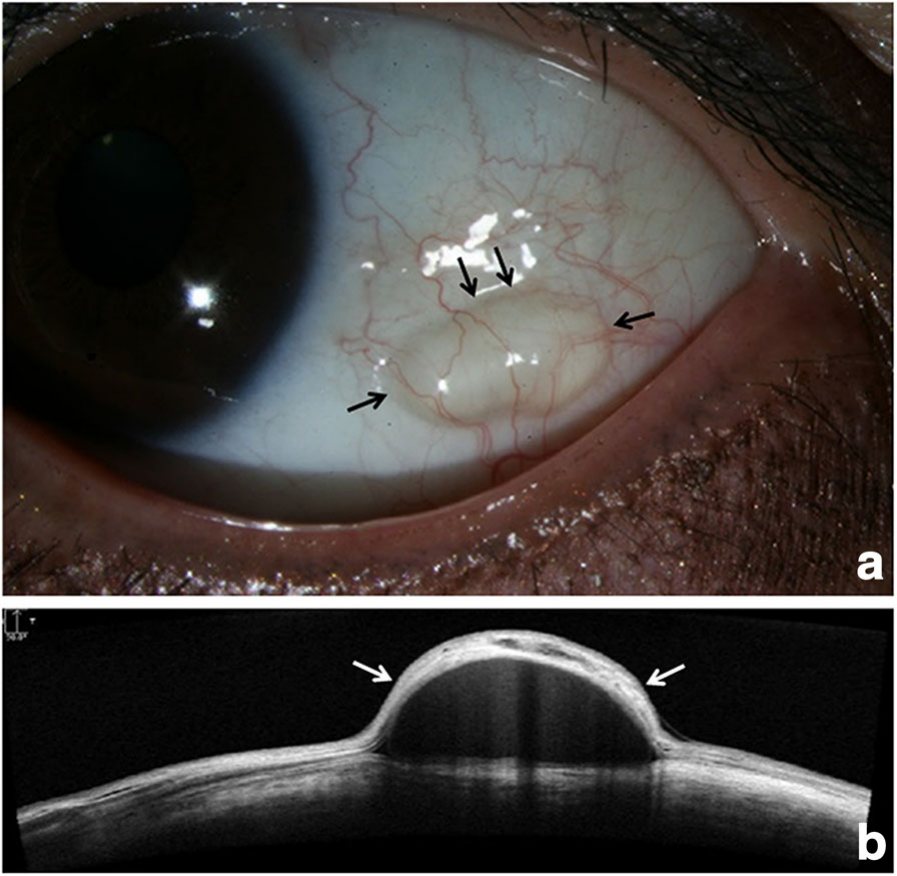
Preoperative findings. **a** Anterior segment photography obtained before the procedure reveals a freely movable conjunctival cyst (*black arrow*); **b** Anterior segment optical coherence tomography (AS-OCT) reveals a subconjunctival cyst filled with homogenous fluid (*white arrow*)

A 50-years-old woman presented with a conjunctival cyst in the left eye that developed 3 months ago. Her medical history was unremarkable, and there was no history of ocular surgery or trauma. Simple aspiration was performed twice (1 and 2 months prior) but was followed by recurrence each time. Slit-lamp examination revealed a 5 mm × 2 mm sized freely movable conjunctival cyst. Anterior segment optical coherence tomography (AS-OCT) demonstrated a subconjunctival cyst filled with homogenous fluid (Fig. 1).

**Fig. 2:**
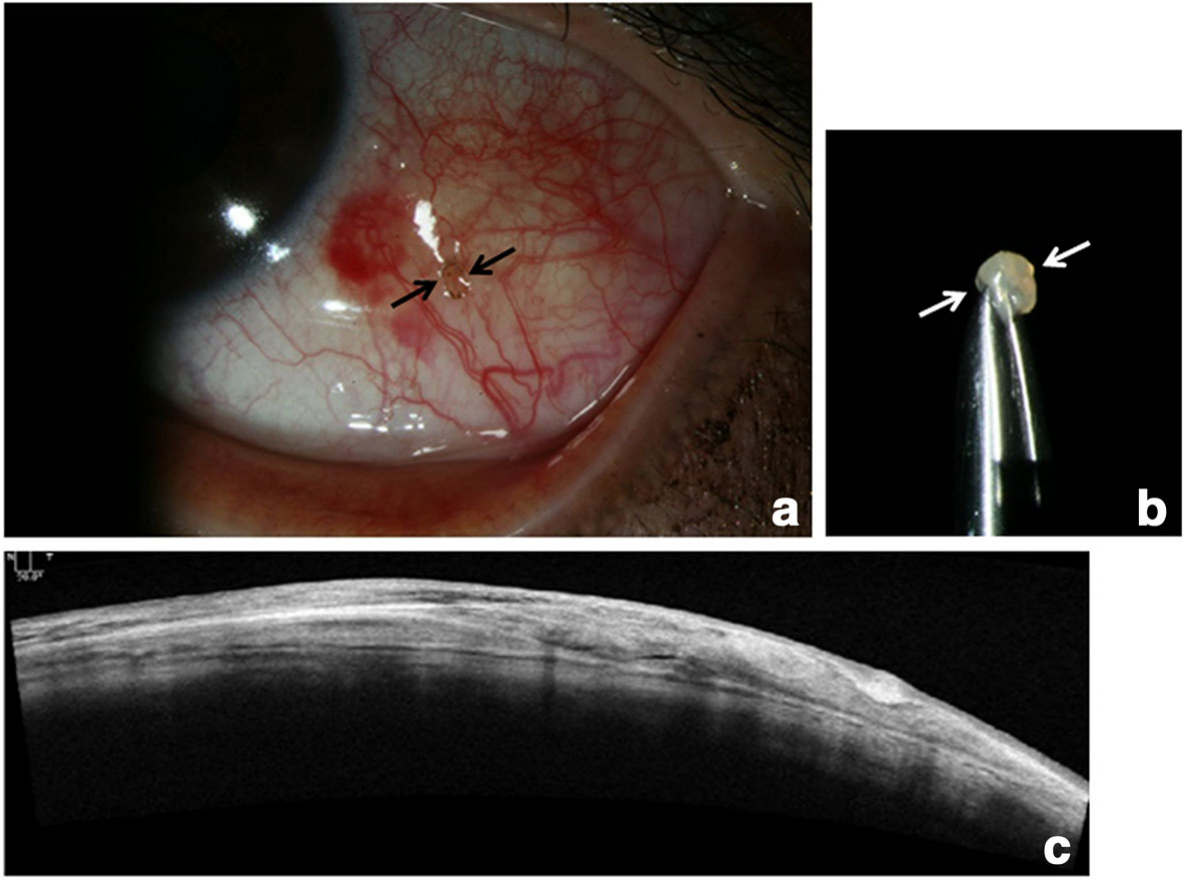
Findings immediately after the procedures. **a** Anterior segment photography obtained immediately after the procedure. Round opening made using PASCAL photocoagulation is visible (*black arrow*); **b** Conjunctival cyst extracted without rupture (*white arrow*); **c** AS-OCT confirms complete removal of the cyst

After obtaining informed consent, cyst removal assisted by PASCAL photocoagulation was performed. After topical administration of 0.5% proparacaine hydrochloride, a round conjunctival opening sized 1 mm × 1 mm was generated using PASCAL photocoagulation (duration 60 ms, power 250–300 mW, spot size 200 μm, 225 shots). A Healon needle was inserted through the opening, and the cyst was isolated by blunt dissection with the needle. The cyst was gently grasped with a non-toothed forceps and extracted through the conjunctival opening made by PASCAL laser. After cyst removal, only a minimal amount of subconjunctival hemorrhage was observed. The cyst was removed without damage to the cyst wall. AS-OCT demonstrated complete removal of the cyst (Fig. 2). The entire procedure was completed within 10 min, and no pain was reported. After treatment, 0.5% topical levofloxacin and 0.1% fluorometholone (qid for each) were prescribed.

**Fig. 3:**
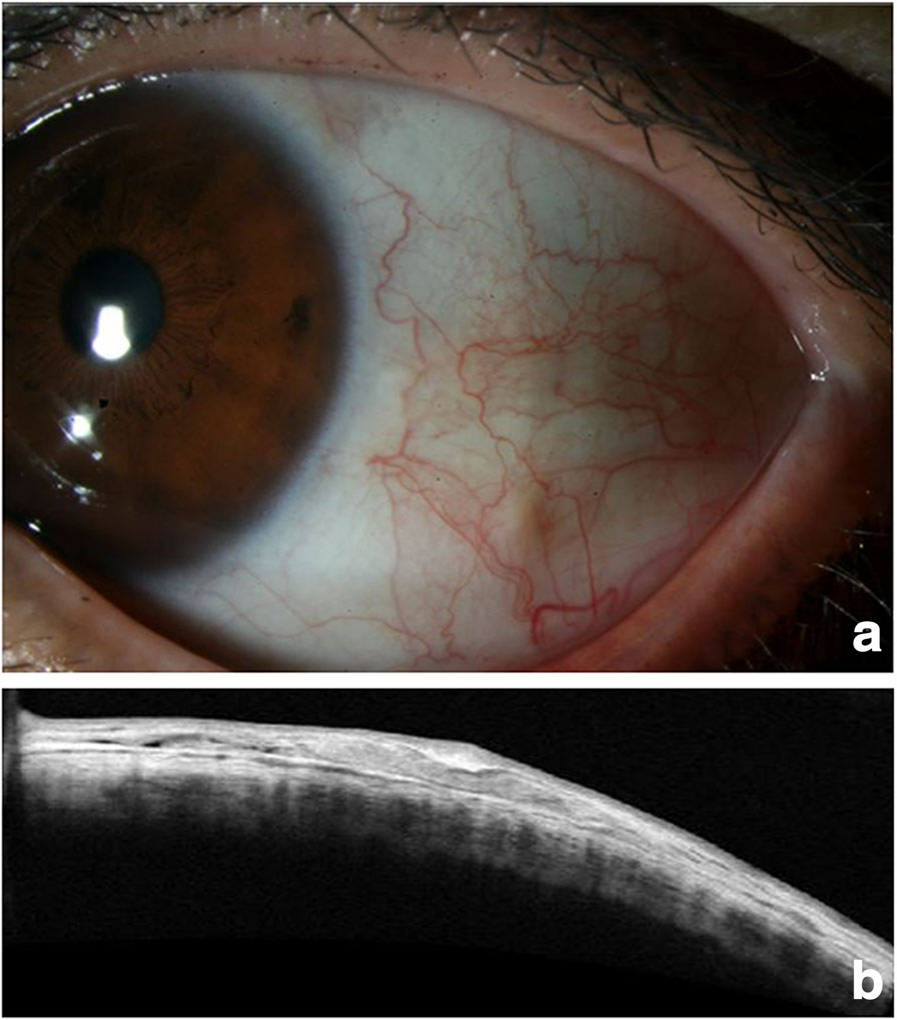
Findings 2 weeks after the procedure. **a** Anterior segment photography obtained 2 weeks after the procedure reveals complete re-epithelialization of the conjunctiva without any complications; **b** AS-OCT indicates complete cyst removal and re-attachment of the subconjunctival space

Two weeks later, the patient reported no discomfort and was satisfied with the cosmetic results. Anterior segment examination revealed complete re-epithelialization of the conjunctiva without any complications or evidence of cyst recurrence, which was also confirmed by AS-OCT (Fig. 3). No evidence of recurrence was detected over the 6-months follow-up period.

## Discussion

Although various techniques have been introduced for the removal of conjunctival cysts, each method has its own limitations. Simple resection is often associated with recurrence caused by incomplete removal of the cyst margin [1]. Visualization of the cyst using tissue dyes is effective for complete resection of the cyst [1, 4]. However, the method requires additional costs for dye [1, 4] and bears the risk of staining normal adjacent tissues or causing cyst rupture by excessive dye injection [1, 2]. Thermal cautery or injection of sclerosing agents can be simple and curative [5, 6], but these methods have the risk of damage to surrounding tissue caused by thermal burn or drug leakage [5, 6]. Collapsing the cyst by applying neodymium-doped yttrium aluminum garnet (Nd:YAG) laser on the cyst surface also has a risk of incomplete cyst removal [7]. Destruction of the cyst using argon laser photoablation can be curative [3]. However, more extensive laser treatment is required to reach the deep-seated cyst base, which can increase pain and treatment time [2]. Therefore, the technique is conceivably used only for small conjunctival cysts [3].

To the best of our knowledge, this report is the first to describe conjunctival cyst removal with the adjunctive use of PASCAL photocoagulation. Laser therapy has recently been replacing surgery for the treatment of ocular surface diseases [2]. PASCAL, a 532-nm frequency-doubled Nd:YAG diode-pumped solid-state laser, reduced pulse duration compared with argon lasers [8]. Reductions in pulse duration are advantageous because this approach increases the predictability of treatment area and decreases pain and surrounding tissue injury through reduced penetration and lateral spread of heat [8]. Thus, PASCAL photocoagulation is expected to be useful in the treatment of ocular surface diseases. Park et al. [8] recently reported the efficacy of PASCAL for conjunctival nevus ablation.

In the present case, we created a conjunctival opening using PASCAL photocoagulation with minimal patient discomfort and collateral injury and attempted to extract the cyst through the small opening because the cyst was freely movable and appeared to have weak attachment to adjacent tissues. Histologically, the cyst cavity is lined by non-keratinizing epithelial cells and goblet cells [9]. Electron microscopy indicates that the epithelial cells were relatively devoid of hemidesmosomes, which may account for the mobility of these cysts [9]. As the cyst has minimal stroma and is filled with mucinous material [9], it may easily change its shape and be extracted through the small opening without rupture, as in the present case. Although a recent study demonstrated that a conjunctival cyst can be removed through a 4.5-mm sized incision [10], this study suggests that it can be extracted through a 1 mm × 1 mm sized opening, even without shrinkage.

The technique introduced in this study is simple and minimally invasive and can be performed in the office setting. The technique can improve patient satisfaction by minimizing pain and post-treatment scar formation. Given that subconjunctival hemorrhage is minimal in our procedure, it can be favorable for patients with increased bleeding tendency. The cost is also one of the benefits of our technique, as it does not require tissue dye or suture material. However, our technique has a limitation in that it can only be applied to freely movable conjunctival cysts. A greater number of different types of conjunctival cyst cases are needed to evaluate the safety and efficacy of the technique.

## Conclusion

PASCAL laser-assisted conjunctival cystectomy can be a safe and efficacious option in the treatment of conjunctival cysts.

## Abbreviations

AS-OCT: Anterior segment optical coherence tomography
Nd:YAG: Neodymium-doped yttrium aluminium garnet
PASCAL: Pattern scan laser
YAG: Yttrium aluminium garnet
